# Normal‐Weight Offspring of Parents With Diet‐Induced Obesity Display Altered Gene Expression Profiles

**DOI:** 10.1002/osp4.70058

**Published:** 2025-02-17

**Authors:** Paul Czechowski, Anne Hoffmann, Sebastian Dommel, Alexander Jais, Matthias Blüher, Nora Klöting

**Affiliations:** ^1^ Helmholtz Institute for Metabolic Obesity and Vascular Research (HI‐MAG) of the Helmholtz Zentrum München at the University of Leipzig and University Hospital Leipzig Leipzig Germany

## Abstract

**Objective:**

A Western diet is associated with obesity, and the link between parental and offsprings' obesity is unclear. Among mice, this study examined how parents' Western diets affect their male offspring's obesity risk. This study further explored whether early exposure to obesogenic diets from either parent influences offsprings' long‐term weight gain.

**Methods:**

Three‐week‐old C57BL6/NTac mice were assigned to a Western diet (WD) or control diet (CD), given from six to 14 weeks old. Adults from these dietary groups were then mated to create four breeding combinations: CD/CD, CD/WD, WD/CD, and WD/WD. Weight gain trajectories were studied in parents (P) and offspring (F1), along with gene expression in four tissues of male offspring. Non‐linear mixed effect modeling and q‐mode PCA were used to assess the influence of sex, litter size, and parental diet on gene expression, before describing gene expression in more detail.

**Results:**

Offsprings' weight gain was mainly influenced by sex and litter size, with no significant impact from parental diet. At the same time, gene expression differences among offspring, particularly between WD/WD and CD/CD offspring, were linked to genes associated with inflammation, stress response, and other obesity‐relevant processes.

**Conclusions:**

Obegenesic diet of two parents with obesity, rather than only one, likely alters the risks of metabolic disease in male mice even at normal weights.

## 1 Introduction

Maternal obesity has been linked to a range of negative health effects in offspring, including an increased risk of obesity [1]. The exact mechanism of passing on obesity from parents to offspring is not yet fully understood, but does not appear to be solely an effect of excessive energy intake among parents and their offspring, but also have an inheritable component [2, 3]. In humans, about 1200 genes are implicated in severe and early‐onset obesity, but the identification of genes promoting obesity will likely not be finished soon [4]. Animal models such as rats [5] and mice [6] are of scientific value to study the heritability and genetic predisposition of obesity among humans [7].

Across mammalian species, obesity may pass on from parents to their offspring by non‐genetic mechanisms, with diet playing a pivotal role in realizing these traits. Two seminal studies [6, 8] found that birth weight differences between offspring of lean dams and dams with obesity disappeared regardless of offspring sex when offspring were nursed by lean dams in rats and mice. Dahlhoff et al. [6] further found female offspring to maintain normal weight regardless of mother's obesity, and daughters of mothers with obesity even had less‐then‐normal body fat. In contrast, sons of mothers with obesity gained more fat and body weight than those of mothers without obesity. Recently [9], found alterations to mice's sperm miRNA mechanistically linked to obesity programming in offspring. Complementing these findings, Chiñas Merlin et al. [10] showed that switching to a standard diet at weaning can attenuate the deleterious effects of long‐term paternal high–caloric intake among male mouse offspring. These findings suggest that among mice, mothers with obesity may pass on obesity traits, particularly to male offspring, or males may be more prone to inheriting obesity than females, with such traits manifesting when being fed a high caloric diet.

Obesity models often use high‐calorie diets, but there is no consensus on the best diet, leading to inconsistent phenotypes, physiological and biochemical parameters, and challenging comparisons across studies [11]. In the real world the Western diet has a strong impact on obesity in humans [12]. As the Western diet also affects mice [13], it can be considered a good choice for studying obesity inheritance in mice to understand the real‐world effects of obesity‐promoting diets in humans.

Although researchers have identified obesity‐related genes in humans [4], the interactions between those remain largely unknown. Utilizing mouse models can provide insights when examining the interplay between diet‐induced obesity and heritable obesity traits. Sex‐specific microarray analysis of rodent adipose tissues revealed distinct gene expression patterns, with males showing a greater propensity for inflammatory responses associated with high‐caloric diets [14]. Considering that other sex‐specific expression differences are also influenced by obesity‐related diseases [15], it seems reasonable to investigate male‐specific expression differences across multiple adipose tissues, including the liver [6], when examining the effects of obesogenic diets.

This study investigated how parental pre‐conceptional exposure to a high‐caloric, high‐cholesterol Western diet affects offsprings' weight gain. Specifically, this work aimed to determine whether such an effect might be sex‐specific to offspring, similar to findings by Dahlhoff et al. [6]. Further, this work aimed to elucidate gene expression changes among male offspring to differentially fed parents by means of DNA microarray analysis of four tissues (inguinal subcutaneous white adipose, interscapular brown adipose, epididymal white adipose, and liver tissue). By investigating sex‐specific body weight changes and associated gene expression, including the liver, this work extends on the work of Dahlhoff et al., Gorski et al., and Chiñas Merlin et al. [6, 8, 10] by accounting for the effects of high‐caloric Western diets.

## 2 Methods

### 2.1 Animal Husbandry and Phenotyping

C57BL6/NTac (BL/6) mice from Taconic were kept in a local animal facility as further described in the Supporting Information S1. All experiments were approved by the Ethics Committee on Animal Health and Care of the State of Saxony (Landesdirektion Sachsen, approval number TVV10/20, T02/2020, TVV31/16) and compliant with European Communities Council Directive of 24th November 1986 (86/609/EEC). Animals had free access to water and food in a climate‐controlled room with a 12‐h light‐dark cycle.

**FIGURE 1:**
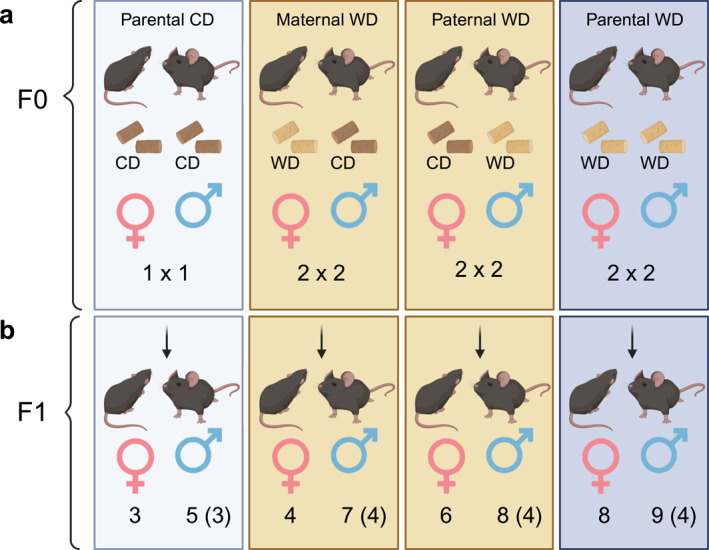
Experimental design. In the F0 generation (a), at 3 weeks of age, seven females and seven male BL/6 mice were distributed between the two groups. One group (three females, three males) was fed a Western control diet (CD; CD.88137, see methods for further details) between 6 and 14 weeks of age. Another group (four females, four males, one female deceased prior to breeding) received the TD.88137 high‐fat, high‐cholesterol diet (henceforth WD; see methods for details) for the same period. Body weights of these F0 mice were measured weekly. At 14 weeks of age, F0 mice were mated, and F1 female and male offspring were raised on a standard maintenance diet, measuring phenotype variables weekly. Sixteen F1 male offspring's gene expression (counts in parentheses) of four tissue types (liver, epididymal visceral, inguinal subcutaneous, and interscapular brown adipose tissue) were then assessed in transcriptome‐wide gene‐level expression profiling using Clariom S mouse assays. Further details on sequenced individuals are provided in SI, in Table S1. Figure created with Biorender.com.

**FIGURE 2:**
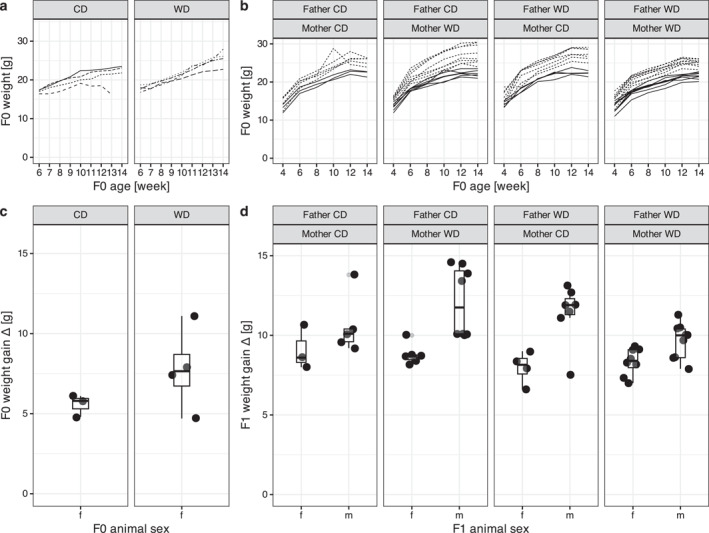
Weight gain of F0 (a) and F1 (b), and weight gain deltas of F0 (c) and F1 (d) over the course of the experiment, by sex and diet. In (a) line types indicate different F0 animals and in (b) line types indicate different F1 sexes. In (c) and (d), the lower and upper hinges correspond to the first and third quartiles. Whiskers extend from the hinge to the largest/smallest value no further than 1.5 inter‐quartile ranges. In F0 (a) the diet, and in F1 offspring the parental diet (b) is indicated with WD for Western diet, and CD for control diet. Note similar extends of *y*‐axis in (a) and (b), and (c) and (d). Weight gain over time for the F1 is shown individually in Supporting Information S1: SI Fig 1 and SI Fig 2 shows modeled population averages by treatment.

To constitute the F0 generation (Figure 1a) seven females and seven male BL/6 mice were randomly distributed between two groups at 3 weeks of age. One F0 group (*n* = 3/3) was fed a Western control diet (henceforth CD; E15720‐04, CD.88137, Sniff, Soest, Germany) with 69 kJ% carbohydrates, 18 kJ% protein, 13 kJ% fat, and a gross energy (GE) of 18.3 MJ/kg and a metabolizable energy (ME) of 15.7 MJ/kg between 6 and 14 weeks of age. The other F0 group (*n* = 4/4) received a high‐fat, high‐cholesterol Western diet (henceforth WD; E15723‐34, TD.88137; Ssniff), containing 43 kJ% carbohydrates, 15 kJ% protein, 42 kJ% fat, with a GE of 21.8 MJ/kg and a ME of 19.1 MJ/kg for the same period. Body weights of F0 mice were measured weekly (Figure 2a).

At 14 weeks of age, F0 female mice were mated to F0 males having been fed with WD and CD (Figure 1) and screened for vaginal plugs mornings and evenings. F0 females remained on their specific diets during pregnancy and lactation.

F1 mice (Figure 1b) were fed a maintenance diet (henceforth MD; E15723‐34, TD.88137; Ssniff), containing 67 kJ% carbohydrates, 24 kJ% protein, 9 kJ% fat, with a GE of 16.2 MJ/kg and a ME of 13.5 MJ/kg, from day 21 after birth until 14 weeks of age (see Figure 1). Body weight of F1 mice was monitored weekly up to 14 weeks of age (Figure 2b; Supporting Information S1: Figure S1). Body composition (percent fat and lean mass) was measured once at the end of this period using a magnetic resonance imaging (MRI) analyzer (Minispec LF50; Bruker, Karlsruhe, Germany) without anesthesia.

At 14 weeks of age, male F1 mice were euthanized, and organs were dissected and weighed. Four tissue types—inguinal subcutaneous white adipose (INGWAT), interscapular brown adipose tissue (IBAT), epididymal white adipose (EWAT), and liver (henceforth LIV; for nomenclature see [16])—were harvested and either processed immediately or snap‐frozen (to −80°C) for analysis.

### 2.2 Analysis of Body Weight Changes

Weights of 21 F1 females and 29 F1 males were measured across six weekly time points (between days 28 and 98) to obtain 300 total weight measurements. Data are summarized in Figure 2b, plotted as trajectories in Supporting Information S1; SI Figure 1, and provided in SOM Table S1.

For F1 mice non‐linear random‐effect models of weight gain over time were encoded in the *R* version (v4.3.3) using package *saemix* (v3.3; [17, 18]). An exponential growth function was used to model weight trajectories.

In this model, F1 weight gain over time was formulated with $y_{ij}=A_{i}\;\left(1-B_{i}e^{{-K}_{i}t_{ij}}\right)+\epsilon_{ij}$ in which, for mouse i, the regression variable was the time (in days) $x_{ij}=\left(t_{ij}\right)$, and curves for each mouse were described by the estimated parameters $\theta_{i}=\left(A_{i},B_{i},K_{i}\;\right)$. The covariate initially added to a null model was sex (f, m). In a subsequent model, litter size was added (between five and nine, see SOM Table S1). In a third model also added were maternal diets (CD, WD), and paternal diets (CD, WD; also compare in Supporting Information S1: Figure S1).

For each model, the conditional Bayesian Information Criterion was obtained, as implemented in *seamix,* and similarity among models was established using likelihood ratio tests. For the model best describing the study data while having the lowest cBIC, model fits were inspected, and parameter estimates were reported. Further details are provided in the Supporting Information S1.

### 2.3 Transcriptome Analysis

RNA isolation, transcriptome data acquisition with Clariom S mouse assays (Applied Biosystems, Waltham, MS, USA) and data pre‐processing are detailed in the SI. Total RNA of 15 F1 male individuals was analyzed (Supporting Information S1: Table S1).

Transcriptome analysis was initiated with Principal Component Analysis (PCA) to find strong tissue‐specific contrasts in gene expression based on litter size, which may have affected subsequent searches for differentially expressed genes (DEGs) based on F1's parents' dietary status. In PCA, expression data was inspected not separated by tissue type. Subsequently, each tissue was analyzed individually, this time using Q‐mode PCA to maximize variance across the first two PCs [19].

To identify transcript information associated with obesity, a subsequent search for differentially expressed genes (DEGs) was conducted, explicitly seeking the existence of 27 such transcripts. A complete listing of these transcripts, alongside references, is provided in the SI.

Based on PCA, DEGs were searched among all four tissues using the contrasts between CD/CD and each of CD/WD, WD/CD, and WD/WD, resulting in a total of six DEG searches of parental dietary combinations for each of the four tissues. For this DEG search, the *R* package *limma* was used. Moderated *t*‐statistics, moderated *F*‐statistics, and log‐odds were calculated using empirical Bayes moderation. DEGs were considered significant only when false discovery rate adjusted *p* values were *p* < 0.05, and the log_2_‐fold change was at least one. DEGs for tissues and contrasts were summarized as tables, and, aiming to be guided by the number of DEGs, Upset plots [20] were used to identify contrasts most relevant between dietary combinations for each tissue.

For tissues and contrasts with the highest numbers of DEGs, *R* package *clusterProfiler* (v4.6.2; [21]) was used for an Over‐Representation Analysis (ORA), so as to identify known biological functions or processes possibly over‐represented among each of the tissue‐specific lists [22]. Over‐Representation Analysis tested the existence of significant (i.e. Benjamini and Hochberg [23],—adjusted *p* < 0.05) associations between DEGs and gene pathways or biological processes (formalized by Gene Ontology terms; [24]).

## 3 Results

### 3.1 Analysis of Body Weight Changes

There was no relevant effect of parental diet on weight gains of offspring. Offsprings' mouse weight gain was best modeled over time when only animal sex and litter size were included (cBIC = 852.2497). Adding parental diet as a covariate resulted in a “worse” model with a substantially higher cBIC (878.5237) which did not significantly differ in its likelihood estimates from the less complex model including only sex and litter size (*p =* 0.42).

The population parameter estimates of weight gain over time for male and female mice offspring, when including parental diets, are shown at the reference level in Supporting Information S1: Figure S2—in the best‐fitting model the growth trajectory for female mice offspring asymptotically approached A at 26.79 g with a standard error (SE) of 1.3 g, for male offspring the corresponding value was 19% higher (SE 1.6%). One more sibling lowered the weight asymptote A by 2.3% (SE 0.6%). Other curve parameters (B,K) were deemed irrelevant by the model.

The obtained model was supported by additional independent Kruskal‐Wallace tests of the effect of parental diet on body weight and MRI‐derived body fat percentages of the male and female offspring at the end of the growth period—in concordance with previous modeling, no significant differences were obvious in those tests (Supporting Information S1: Figure S3).

### 3.2 Transcriptome Analysis

PCA of expression data across all tissues in unison indicated that only tissue type, but neither parental (F0) diets, nor F1 litter size significantly arranged samples along the first two PCs (Supporting Information S1: Figure S4). Likewise, neither litter size, nor body weight appeared to influence expression data clustering in Q‐mode PCA (Supporting Information S1: Figures S5–S8).

Normalized expression values of existent obesity related genes in each of the four tissues, and within each tissue for each relevant contrast, are listed in Supporting Information S1: Figure S9. None of these genes were later discovered among significant DEGs.

**FIGURE 3:**
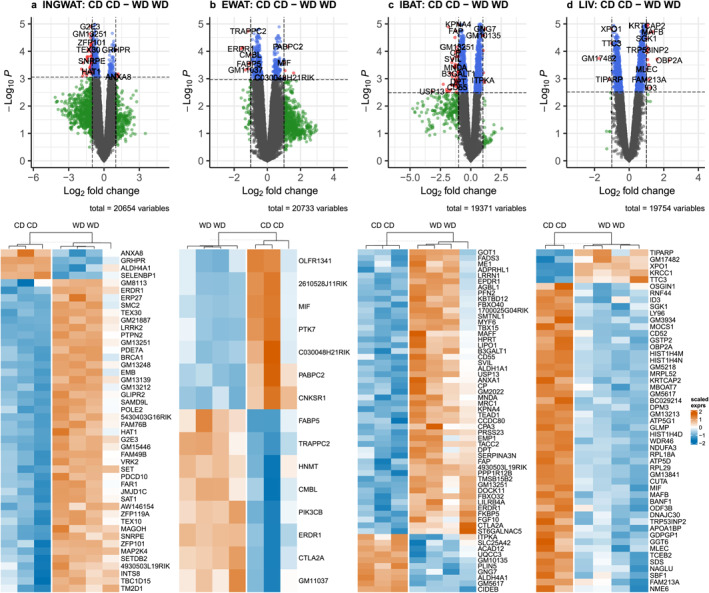
Significant associations of differentially expressed genes (DEGs) in (a) inguinal subcutaneous adipose tissue (INGWAT), (b) epididymal visceral adipose tissue (EWAT), (c) interscapular brown adipose tissue (IBAT), and (d) liver tissue (LIV), as contrasted by biparental Western diets (WD/WD) and biparental Western control diet (CD/CD). For DEG analysis, moderated *t*‐statistics, moderated *F*‐statistics and *log*‐odds were calculated by empirical Bayes moderation. Volcano plots show DEGs with significance above *p* = 0.05 and a log_2_ fold‐change cut‐off of 1.0 in red, and those genes are presented again in the heat maps.

**FIGURE 4:**
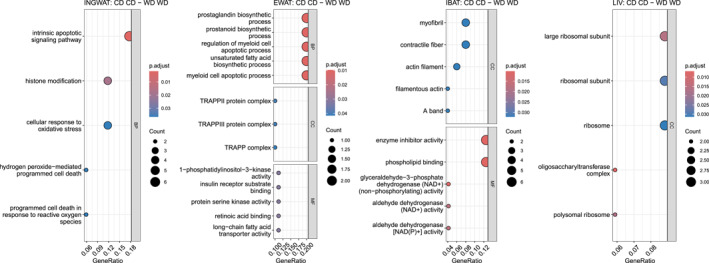
Significant associations of DEGs (see Fig 3) with Gene Ontology terms (GO) in Over‐Representation Analysis [22] of inguinal subcutaneous adipose tissue (INGWAT), epididymal visceral adipose tissue (EWAT), interscapular brown adipose tissue (IBAT), and liver tissue (LIV). Where discovered, plots are split into biological processes (BP), cellular components (CC), and molecular functions (MF), which are further discussed in the main text. For the underlying tables consult the SI.

Differentially expressed genes (DEGs) for each of the four tissues and relevant contrasts are summarized as Upset plots in Supporting Information S1: Figures S10 and listed in SOM Table S2. Among all four tissues, the DEG count was always highest for the parental dietary contrast of CD/CD against WD/WD (INGWAT: 46, EWAT: 11, IBAT: 44, LIV 33). Volcano plots and heat maps for DEGs (CD/CD vs. WD/WD) of each analyzed tissue are shown in Figure 3, top and middle panels. Significant associations of DEGs with each of the for tissues with GO terms are summarized in Figure 4.

Most significantly enriched GO terms in INGWAT and WD/WD offsprings (Figure 4, SOM Table S3) included biological processes related to intrinsic apoptotic signaling pathways, hydrogen peroxide‐mediated programmed cell death, and programmed cell death in response to reactive oxygen species, associated with upregulation of PDCD10 (Programmed Cell Death 10) and MAP2K4 (Mitogen‐Activated Protein Kinase 4), respectively. Likewise, upregulated LRRK2 (Leucine Rich Repeat Kinase 2) was associated with many enriched GO terms related to cellular responses to reactive oxygen species.

Most significantly decreased GO terms in EWAT in WD/WD offsprings (Figure 4, SOM Table S4) included biological pathways related to prostaglandin biosynthetic processes, prostanoid biosynthetic processes, and unsaturated fatty acid biosynthetic processes, identified by downregulation of FABP5 (Fatty Acid Binding Protein 5) and MIF (Macrophage Migration Inhibitory Factor). Enriched, on the contrary, were myeloid cell apoptotic processes, myeloid cell apoptotic processes, and negative regulation of intrinsic apoptotic signaling pathways, evident through upregulation of PIK3CB (Phosphatidylinositol‐4,5‐Bisphosphate 3‐Kinase Catalytic Subunit Beta).

Most significantly enriched GO terms in IBAT, enriched in WD/WD offsprings (Figure 4, SOM Table S5), were associated with the upregulation of PPP1R12 B (Protein Phosphatase 1 Regulatory Subunit 12B) and included metabolic functions related to enzyme inhibitor activity, and cytoskeletal components. Furthermore, a pathway involving glyceraldehyde‐3‐phosphate dehydrogenase activity, and others, were highly enriched linked to upregulation of ALDH1A1 (Aldehyde Dehydrogenase 1 Family Member A1) and downregulation of ALDH1A1 (Aldehyde Dehydrogenase 4 Family Member A1).

Most significantly enriched GO terms in LIV and downregulated in WD/WD offsprings (Figure 4, SOM Table S6) included cellular oligosaccharyl‐transferase complex components due to downregulation of KRTCAP2 (Keratinocyte Associated Protein 2) and MLEC (Malectin). All other GO terms were enriched linked to the downregulation of RPL29 (60S Ribosomal Protein L29), RPL18 A (Ribosomal Protein L18a), and MRPL52 (Mitochondrial Large Ribosomal Subunit Protein ML52).

## 4 Discussion

A mouse model with diet‐inducible obesity was used to investigate the effect of parental high‐caloric Western diet on transcriptional changes in metabolic organs of male mice. While the body weight of the offspring was influenced by mice’ sex and litter size as expected [25], a weight or body fat percentage change related to parental diet was not detected. The most significant transcriptomic differences were observed between male mice whose parents were both fed a Western diet (WD/WD) and those whose parents were fed a control diet (CD/CD). Those contrasting transcriptomic changes did not include 27 transcripts identified by other authors as associated with obesity or co‐morbidities; instead, new transcripts were identified.

PCA indicated that neither litter nor access to parental diet had a strong effect on transcriptomes. In rodent studies related to metabolic diseases, observations and measurements are confounded by variable litter sizes [25]. In this study, post‐weaning weights were not adjusted for their linear relationship with litter size, as theoretically possible based on literature consultation [26], but those authors used different mouse strains and diets than strains used here, and such a mathematical correction would not have impacted transcriptomic effects. Therefore, the experimental design pursued in this study does not eliminate the possibility that litter size may have confounded the observed effects on the analyzed transcriptomes, just as it could not account for weight differences between individual mice. Similarly, while it was never observed, it cannot be completely ruled out that some pups may have accessed their mothers' high‐calorie diets between days 16 and 21 [27]. In the end, PCA and modeling of weight gain trajectories indicated that litter size did not affect measurements, and the study presented here is among the few to consider litter size at all [25]. The PCA also explained why relatively few DEGs were found—with in each litter expression signals were heterogeneous (Supporting Information S1: Figures S4–S8).

Parental diet appears to influence both liver gene expression and fat deposition patterns in offspring, with distinct effects on metabolic health. Although the number of F0 breeding pairs may be relatively small when compared to other studies to assess gene expression [28], the parental diet appeared to have a strong impact on some of F1's gene expression related to liver function and fat storage. The most notable differences in gene expression were observed in offspring whose parents followed a Western diet (WD/WD) compared with those whose parents were on a control diet (CD/CD). Significant changes in liver gene expression (Figs 3, 4) point to an association between Western diet consumption and alterations in lipid metabolism, increased liver fat, and changes in body fat distribution, all of which are known to influence metabolic health [28, 29, 30]. Interestingly, upper body fat deposits, namely INGWAT and IBAT, showed more transcriptomic differences than lower body fat deposits, namely EWAT (Figure 3, Supporting Information S1: Figure S9). This distinction is important, as body fat distribution plays a critical role in metabolic health outcomes: upper body or visceral fat is associated with greater metabolic complications, while lower body fat is typically linked to fewer adverse metabolic effects [31, 32]. As detailed further below, these findings suggest that changes in gene expression in different body fat depots could have detrimental effects on health, particularly in the context of a Western diet.

The observed gene expression in the liver hints at increased cellular stress and possible regulation of inflammatory processes in response to parental Western Diet. The downregulation of KRTCAP2 and MLEC in the liver is not well understood, although high‐fat diets have been shown to alter hepatic gene expression, including downregulating genes involved in lipid metabolism and transport [33], as well as changes in the expression of drug‐metabolizing enzymes [34]. On the contrary, it is reasonable to assume the upregulation of ribosomal proteins could be caused by high‐fat diets, as those have been shown to affect expression of miRNAs [35]. Hence, upregulation of RPL genes involved in protein synthesis could thus be a response to increased cellular stress, a need for increased protein synthesis under the influence of a Western diet, or immigration of additional cells into the organ due to inflammation.

In INGWAT the most prominently enriched GO terms (Figure 4) suggest the presence of inflammatory processes and cell death in a context of obesity and a high caloric diet. No evidence of a link between a Western diet and the upregulation of GO‐term associated PDCD10, MAP2K4, and LRRK2 could be found. However, the involvement of MAP2K4 and LRRK2 in stress response and signal transduction suggests a potential connection, as Western diets are known to induce metabolic stress and inflammation [12]. MAP2K4 (also known as MKK4) is a kinase involved in the JNK signaling pathway and has been implicated in inflammation and stress responses [36]. LRRK2 has been shown to affect signal transduction pathways, including the ERK module, and may influence autophagy [37]. Interestingly, the ERK pathway is known to be upregulated in adipose tissue in response to certain stimuli, including diet‐induced obesity [38]. Lastly, Feng et al. [39] investigated whether adipocyte cell death is a consequence of macrophage inflammation, or vice versa, in the context of a high‐fat diet, and suggested that a adipocyte cell death is an intrinsic response independent of macrophage infiltration, which may explain the upregulation of PDCD10.

In IBAT, GO overrepresentation analysis indicated ongoing compensatory processes counteracting excessive weight gain. Upregulation of PPP1R12 B and ALDH1A1 in IBAT associated with high caloric intake could be a compensatory response to increased energy intake aimed at enhancing thermogenesis and preventing excessive weight gain. Conversely, downregulation of other genes like ALDH4A1 might reflect a disruption in metabolic pathways [40].

Gene expression in EWAT, like in IBAT, may represent regulatory processes counteracting inflammation of fat tissue when facing energy influx. Downregulated FABP5 in EWAT associated with a Western diet may suggest a modulation of lipid metabolism in response to dietary fat composition. FABP5, as an intracellular chaperone of fatty acids, is involved in the regulation of lipid metabolism and cell growth, and its expression is increased in various cancers [41]. Furthermore, MIF is a pro‐inflammatory cytokine involved in the regulation of insulin resistance and obesity [42]. A Western diet rich in fat and sugar may trigger an inflammatory response in adipose tissue, which could lead to downregulation of MIF as a compensatory mechanism to mitigate inflammation. This hypothesis is supported by the observation that MIF mRNA expression was decreased in the epididymal fat of rats with obesity and diabetes [43]. Lastly, PIK3CB is a catalytic subunit of the enzyme PI3K, which upon activation by growth factors, cytokines, and other extracellular stimuli, initiates a cascade of downstream effects that regulate cell growth, differentiation, survival, and function [44]—upregulation of PIK3CB could thus indicate ongoing activity in EWAT due to high caloric intake.

The direct translation of the findings obtained here to humans remains difficult for the time being. Comparison of GO terms related to fat tissue expression in humans with obesity [45] and the mouse‐derived terms isolated here show only few similarities. Specifically, both sets of terms, including the human GO terms, listed myeloid cell activity as enriched. Myeloid cell activity may be enriched due to inflammatory responses in fat tissues. However, beyond these similarities, it is likely that there is little overlap, as the presented comparison involves two different species, and unlike the subjects in Lu et al. [45], our (F1) mice did not have obesity.

Lastly, Næss et al. and Fan and Zhang [46, 47] highlight strong associations between parental obesity and higher offspring BMI, suggesting that genetic and environmental factors inherited from parents with obesity increase the risk of obesity in offspring. It is conceivable that the gene expression patterns observed here are indicative of offsprings to parents fed with Western diets being “primed” to suffer obesity once they have access to an obegenesic diet, even when such phenotypic changes are not apparent on a maintenance diet.

## 5 Conclusion

Offspring with biparental obesity exhibited significant changes in gene expression among all adipose tissues and the liver, even though no weight change was apparent. Gene expression in adipose and liver tissue of male offspring observed here may support, and extend on, findings by Dahlhoff et al. [6] by corroborating an influential role of parents with obesity on negative obesity‐related outcomes. While the molecular physiological mechanisms underlying embryonic stages of obesity were not investigated here (unlike in [9]), insight into the impact of parental diet on offspring risks for obesity and metabolic disease is provided—male mice with both parents exposed to an obesogenic Western diet exhibited strong changes in gene expression without weight change, suggesting that the exposure to parental western diet may amplify the risk for obesity and metabolic disease which is not evident by weight measurement.

## Author Contributions

Conceptualization: N.K., P.C.; data curation: P.C., S.D., A.H.; formal analysis: P.C., A.H.; funding acquisition: N.K., M.B.; investigation: P.C., N.K., A.H., S.D.; methodology: P.C., A.J., N.K., A.H.; project administration: P.C., N.K.; software: P.C., A.H.; validation: P.C., A.J.; visualization: P.C.; writing–original draft: P.C.; writing–review and editing: P.C., A.J., N.K., M.B., A.H. All authors have read and agreed to the published version of the manuscript.

## Conflicts of Interest

M.B. received honoraria as a consultant and speaker from Amgen, AstraZeneca, Bayer, Boehringer‐Ingelheim, Lilly, Novo Nordisk, Novartis, and Sanofi. All other authors declare no conflicts of interest. The funders had no role in the design of the study, in the collection, analyses, or interpretation of data, in the writing of the manuscript, or in the decision to publish the results.

## Supporting information

Supporting Information S1

Table S1

Table S2

Table S3

Table S4

Table S5

Table S6

## Data Availability

Microarray data have been deposited in the ArrayExpress database [48] at EMBL‐EBI (www.ebi.ac.uk/arrayexpress) with accession E‐MTAB‐13493. Analysis code can be reviewed at https://github.com/macrobiotus/mouse_mating, and the latest stable release is available via 10.5281/zenodo.10040371. Tables summarizing DEGs and GO analysis are provided as Supplemental Online Materials in Microsoft Excel format.
