# Supplementary material for: Normal‐Weight Offspring of Parents With Diet‐Induced Obesity Display Altered Gene Expression Profiles
Source: Obes Sci Pract. 2025 Feb 17;11(1):e70058. doi: 10.1002/osp4.70058 (PMC11832301; doi:10.1002/osp4.70058)
Supplement: Supplementary file 1 — Supporting Information S1 [file OSP4-11-e70058-s004.docx]

Normal-weight offspring of parents with diet-induced obesity display altered gene expression profiles – Supplemental Information

# Supplemental Methods

## Animal husbandry and phenotyping

Male and female C57BL6/NTac mice were purchased from Taconic (Taconic, Denmark). Mice were maintained under specific pathogen-free conditions at 23 °C, 50% humidity and with a 12 h light/dark cycle (lights on at 6 AM). All animals had free access to their specific rodent diet and water *ad libitum*. Mice of both sexes were used for experiments.

## Analysis of body weight changes

We measured weights of 21 females and 29 males across six time points (between days 28 and 98) to obtain 300 weight measurement in total. Among the 50 F1 mice litter size ranged from five to nine with mean 7.5). Data are summarized in Fig. 2b (main text), plotted as trajectories, with parental diets in SI Fig 1, and provided in SOM table 1.

Among female offspring, body weight ranged from 11 g to 23.8 g across all measurement days, fathers of seven received control diet and of 14 received high caloric diet, mothers of nine received control diet and of twelve received high caloric diet. Female offspring’s mean litter size was 7.5.

Among males, body weight ranged from 12.3 g to 30.5 across all measurement days, fathers of twelve received control diet and of 17 received high caloric diet, mothers of 13 received control diet and of 29 received high caloric diet. Male offspring’s mean litter size was 7.3.

Non-linear random-effect models of weight gain over time for these data were encoded in version v4.3.3, using package *saemix* v3.3. After considering Richards and Gomportz functions (compare Tjørve and Tjørve 2017), we settled on using an exponential growth function to model F1 mice weight trajectories. We confirmed our choices using null models, and the Akaike Information Criterion (AIC) implemented in the *seamix* package (Akaike 1974; Comets, Lavenu, and Lavielle 2017).

The exponential growth model we selected described the weight gain over time with $y_{ij}=A_{i} \left( 1-B_{i}e^{{-K}_{i}t_{ij}} \right)+\epsilon_{ij}$in which, for mouse $i$, the regression variable was the time (in days) $x_{ij}=(t_{ij})$, and curves for each mouse were described by the estimated parameters $\theta_{i}=(A_{i},B_{i} ,K_{i} )$. The covariate added initially to our null model was sex (f, m). In a subsequent model we added litter size (between five to nine). In a third model we also added mothers’ diets (control, high-caloric) and fathers’ diets (control, high-caloric).

For each model we obtained the conditional Bayesian Information Criterion (cBIC; Schwarz 1978) as implemented in *seamix*, and tested for similarity among models using likelihood ratio tests (Shapiro and Wilk 1965). For the model describing our data best while, having the lowest cBIC, we inspected the model fit and report parameter estimates here.

## Transcriptome analysis

We isolated total RNA from frozen tissue using guanidinium thiocyanate and phenol (TRIzol, Life Technologies, Grand Island, NY, USA) according to the manufacturer’s instructions. Expression levels of over 20 000 genes were then assessed in transcriptome-wide gene-level expression profiling using Clariom S mouse assays (Applied Biosystems, Thermo Fisher Scientific, Waltham, MS, USA). After sequencing, we background-corrected and quantile-normalized expression data using the Robust Multichip Average provided by R package *oligo* (v1.60.0; Irizarry et al. 2003; Carvalho and Irizarry 2010). Control samples were removed, and genes expressed below a threshold of four in at least as many arrays as the smallest experimental group, based on their transcript median intensities, were excluded from the normalized data (Klaus and Reisenauer 2018). Packages *Biobase* (v2.56.0; Gentleman et al. 2023), oligo and *arrayQualityMetrics* (v3.52.0; Kauffmann and Huber 2023) were used for quality control of raw and normalized data.

After consulting Mahmoud, Kimonis, and Butler (2022), Hua et al. (2023), Dahlman and Arner (2010), and Ginete et al. 2021), we screened all tissue specific data sets for the existence of transcripts for Adiponectin (ADIPOQ), Adrenoceptor β 2 (ADRB2), Alstrom Syndrome Protein 1 (ALMS1), ADP Ribosylation Factor Like GTPase 15 (ARL15), Bardet-Biedl Syndrome 9 (BBS9), Brain Derived Neurotrophic Factor (BDNF), CASK Interacting Protein 2 (CASKIN2), Early Growth Response 2 (EGR2), Neuropeptide FF Receptor 2 (GPR74), Gremlin 1, DAN Family BMP Antagonist (GREM1), Insulin Like Growth Factor 2 Receptor (IGF2R), Insulin Receptor Substrate 1 (IRS1), Intelectin 1 (ITLN1), Leptin (LEP), (LEPR), LDL Receptor Related Protein 2 (LRP2), Melanocortin 4 Receptor (MC4R), Neuropeptide Y Receptor Y1 (NPY1R), Neurotrophic Receptor Tyrosine Kinase 2 (NTRK2), Proprotein Convertase Subtilisin/Kexin Type 1 (PCSK1), Proopiomelanocortin (POMC), Peroxisome Proliferator Activated Receptor Gamma (PPARG), Replication Initiator 1 (REPIN1), SIM BHLH Transcription Factor 1 (SIM1), SPG11 Vesicle Trafficking Associated, Spatacsin (SPG11), Sterol Regulatory Element Binding Transcription Factor 1 (SREBP1), and YY1 Transcription Factor (YY1; gene names and abbreviations correspond with GenCards; Rebhan et al. 1997).

To increase signal-to-noise ratios we considered array weights in *limma*’s (v3.52.4; Ritchie et al. 2015) linear models. Thresholds for identification of differentially expressed genes (DEGs) were parametrized with a false discovery rate (FDR; Benjamini and Hochberg, 1995) < 0.05 and an absolute log_2_-fold change (FC) ≥ 1. Moderated *t*-statistics, moderated *F*-statistics, and log-odds were calculated using empirical Bayes moderation (Smyth 2004).

# Supplemental Results

## Animal keeping and phenotype measurements

SI Table 1: F1 Individuals used for RNA sequencing, including grouping variables related to parental diet. Only males were used for sequencing. Also see number counts in parentheses in Fig 1.

| **Animal ID** | **Mothers Diet** | **Fathers Diet** | **Litter Size** |
| --- | --- | --- | --- |
| A339 | CD | CD | 8 |
| A340 | CD | CD | 8 |
| A342 | CD | CD | 8 |
| A298 | CD | WD | 7 |
| A299 | CD | WD | 7 |
| A300 | CD | WD | 7 |
| A305 | CD | WD | 7 |
| A285 | WD | CD | 5 |
| A346 | WD | CD | 6 |
| A347 | WD | CD | 6 |
| A349 | WD | CD | 6 |
| A312 | WD | WD | 8 |
| A313 | WD | WD | 8 |
| A320 | WD | WD | 9 |
| A321 | WD | WD | 9 |

## Analysis of weight-related phenotype changes

SI Figure 1: Weight gain of 50 mice (F1) with differentially fed parents (F0), used to model weight gain trajectories. We measured weights of 21 F1 females and 29 F1 males across six time points (between days 28 and 98) to obtain 300 weight measurement in total. Among F1 females, body weight ranged from 11 g to 23.8 g across all measurement days, fathers of seven had received control diet (CD) and of 14 had received high caloric diet (WD), mothers of nine had received CD and of twelve had received WD. Among F1 males, body weight ranged from 12.3 to 30.5 g across all measurement days, fathers of twelve had received CD and of 17 had received WD, mothers of 13 had received CD and of 29 received had WD. Mice weight gain was best modelled over time when including animal sex and litter size only, parental diet did not have a marked effect. For litter sizes refer to SI Table 1.

**SI Figure 2:** Population parameter estimates of weight gain over time for male and female mice offspring F1, when including parental diets, at reference level. Parameter likelihood estimated of this model did not significantly differ from those obtained when only including litter size, so that we concluded that there is no relevant effect of parental diet on offspring weight gain.

**SI Figure 3:** Kruskal Wallace tests (Kruskal and Wallis 1952) of the effect of parental diet on body weight (a, b) and body fat percentage (c, d) of the female (a, c) and male (b, d) offspring at the end of the growth period. No significant differences were obvious depending on parental diet, neither for body weights, no body fat percentage. Visualisation created using R package *ggstatsplot 0.12.3* (Patil 2021).

## Transcriptome analysis

PCA results of expression across all tissues in unison indicated that only tissue type, but neither parental (F0) diets, nor F1 litter size significantly arranged samples along the first PC (SI Fig. 3). IBAT and LIV remained remarkably distinct in along the first and second principal components (PC), EWAT and INGWAT appeared similar along the first two PCs.

When each tissue was inspected individually, first and second PC scores remained too low to meaningfully interpret results, necessitating a switch to Q-mode PCA. There, in each of the four tissue types, neither parental diet, nor litter size appeared to have influence on PC1, and all variation was captured by the second PC. (SI Figs 4–7). Neither in IBAT (SI Fig 4), LIV (SI Fig 5), EWAT (SI Fig 6), nor INGWAT (SI Fig 7) sample grouping was obvious.

**
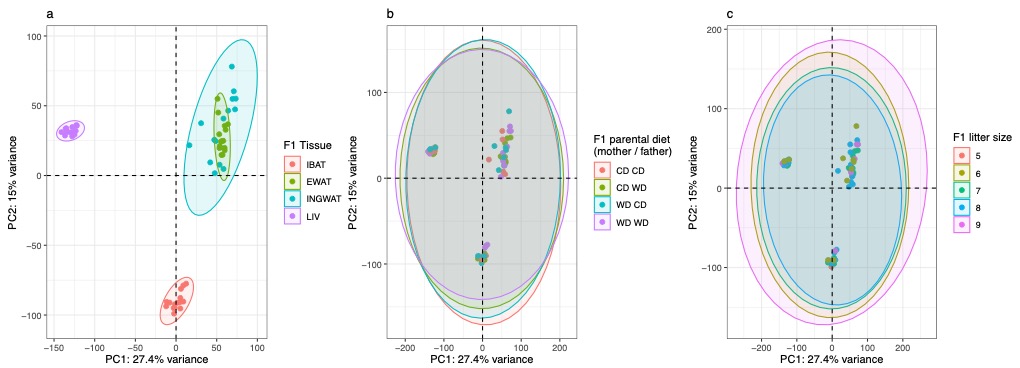
**

**SI Figure 4:** Principal components analysis (PCA) of combined F1 expression data for four tissue types (epididymal visceral, EWAT; inguinal subcutaneous, INGWAT; liver, LIV; and interscapular brown adipose tissue, IBAT). (a) Coloured by tissue type, (b) by F0 diet (Western diet: WD, and control diet: CD), and (c) by litter size. Shown are the most-loaded first two PCs expressing 42.4% of total variation. PCA presented in regular (R) mode due to sufficient variation on the first PCs (Camargo 2022).

**SI Figure 5:** Principal components analysis (PCA) of F1 interscapular brown adipose tissue (IBAT) expression data, presented in q-mode to maximize explanatory power (Camargo 2022) on the first principal components (PCs). Shown are parental dietary treatments (mothers’ and fathers’ diets with Western diet: WD, and control diet: CD) in (a), and by litter size in (c). Shown are the most-loaded first two PCs expressing 95.5% of total variation. Square cosine (Cos 2) value indicates quality of representation, darker vectors deviate from the PC1/2 plane.

**SI Figure 6:** Principal components analysis (PCA) of F1 liver tissue (LIV) expression data, presented in q-mode to maximize explanatory power (Camargo 2022) on the first principal components (PCs). Shown are parental dietary treatments (mothers’ and fathers’ diets with Western diet: WD, and control diet: CD) in (a), and by litter size in (c). Shown are the most-loaded first two PCs expressing 98.9% of total variation. Square cosine (Cos 2) value indicates quality of representation, darker vectors deviate from the PC1/2 plane.

**SI Figure 7:** Principal components analysis (PCA) of F1 epigonodal visceral adipose tissue (EWAT) expression data, presented in q-mode to maximize explanatory power (Camargo 2022) on the first principal components (PCs). Shown are parental dietary treatments (mothers’ and fathers’ diets with Western diet: WD, and control diet: CD) in (a), and by litter size in (c). Shown are the most-loaded first two PCs expressing 98.6% of total variation. Square cosine (Cos 2) value indicates quality of representation, darker vectors deviate from the PC1/2 plane.

**SI Figure 8:** Principal components analysis (PCA) of F1 inguinal subcutaneous (INGWAT) expression data, presented in q-mode to maximize explanatory power (Camargo 2022) on the first principal components (PCs). Shown are parental dietary treatments (mothers’ and fathers’ diets with Western diet: WD, and control diet: CD) in (a), and by litter size in (c). Shown are the most-loaded first two PCs expressing 98.3% of total variation. Square cosine (Cos 2) value indicates quality of representation, darker vectors deviate from the PC1/2 plane.

**SI Figure 9:** Mean gene expression of 27 obesity-associated genes mentioned in Mahmoud, Kimonis, and Butler (2022), Hua et al. (2023), Dahlman and Arner (2010), or Ginete et al. 2021) in four tissues types (epididymal visceral, EWAT; inguinal subcutaneous, INGWAT; liver, LIV; and interscapular brown adipose tissue, IBAT) and the respective dietary contrasts. Parental diets are indicated for mother and father, respectively, with CD for control diet and WD for Western Diet. Shown are row-centered expression values. For a complete listing of transcript names see the supplemental methods.

**SI Figure 10:** Summary of Differentially Expressed Genes (DEGS). Shown are counts of differentially expressed genes within and across analyzed contrasts, contrast themselves were defined by maternal and paternal diets. All tissue combined shown in (a). Inguinal subcutaneous white adipose (INGWAT) only yielded results for one contrast (WD / WD vs. CD / CD, with 46 DEGs), it was hence omitted from figure. Epididymal white adipose (EWAT) shown in second plot (b), interscapular brown adipose tissue (IBAT) shown in (c), liver tissue (LIV) shown in (d). Figure generated with R package UpsetR (Conway, Lex, and Gehlenborg 2017; Lex et al. 2014).

# References

Akaike, Hirotugu. 1974. “A New Look at the Statistical Model Identification.” *IEEE Transactions on Automatic Control* 19 (6): 716–23. https://doi.org/10/d98qkw.

Benjamini, Yoav, and Yosef Hochberg. 1995. “Controlling the False Discovery Rate: A Practical and Powerful Approach to Multiple Testing.” *Journal of the Royal Statistical Society: Series B (Methodological)* 57 (1): 289–300. https://doi.org/10.1111/j.2517-6161.1995.tb02031.x.

Camargo, Arley. 2022. “PCAtest: Testing the Statistical Significance of Principal Component Analysis in R.” *PeerJ* 10 (February):e12967. https://doi.org/10.7717/peerj.12967.

Carvalho, Benilton S., and Rafael A. Irizarry. 2010. “A Framework for Oligonucleotide Microarray Preprocessing.” *Bioinformatics* 26 (19): 2363–67. https://doi.org/10.1093/bioinformatics/btq431.

Comets, Emmanuelle, Audrey Lavenu, and Marc Lavielle. 2017. “Parameter Estimation in Nonlinear Mixed Effect Models Using Saemix, an R Implementation of the SAEM Algorithm.” *Journal of Statistical Software* 80 (August):1–41. https://doi.org/10.18637/jss.v080.i03.

Conway, Jake R., Alexander Lex, and Nils Gehlenborg. 2017. “UpSetR: An R Package for the Visualization of Intersecting Sets and Their Properties.” Edited by John Hancock. *Bioinformatics* 33 (18): 2938–40. https://doi.org/10/gbjxr9.

Dahlman, Ingrid, and Peter Arner. 2010. “Chapter 3 - Genetics of Adipose Tissue Biology.” In *Progress in Molecular Biology and Translational Science*, edited by Claude Bouchard, 94:39–74. Genes and Obesity. Academic Press. https://doi.org/10.1016/B978-0-12-375003-7.00003-0.

Gentleman, R., V. Carey, M. Morgan, S. Falcon, Haleema Khan, and Bioconductor Package Maintainer. 2023. “Biobase: Biobase: Base Functions for Bioconductor.” Bioconductor version: Release (3.17). https://doi.org/10.18129/B9.bioc.Biobase.

Ginete, Catarina, Bernardo Serrasqueiro, José Silva-Nunes, Luísa Veiga, and Miguel Brito. 2021. “Identification of Genetic Variants in 65 Obesity Related Genes in a Cohort of Portuguese Obese Individuals.” *Genes* 12 (4): 603. https://doi.org/10.3390/genes12040603.

Hua, Yuchen, Danyingzhu Xie, Yugang Zhang, Ming Wang, Weiheng Wen, and Jia Sun. 2023. “Identification and Analysis of Key Genes in Adipose Tissue for Human Obesity Based on Bioinformatics.” *Gene* 888 (December):147755. https://doi.org/10.1016/j.gene.2023.147755.

Irizarry, Rafael A., Bridget Hobbs, Francois Collin, Yasmin D. Beazer‐Barclay, Kristen J. Antonellis, Uwe Scherf, and Terence P. Speed. 2003. “Exploration, Normalization, and Summaries of High Density Oligonucleotide Array Probe Level Data.” *Biostatistics* 4 (2): 249–64. https://doi.org/10.1093/biostatistics/4.2.249.

Kauffmann, Audrey, and Wolfgang Huber. 2023. “arrayQualityMetrics: Quality Metrics Report for Microarray Data Sets.” Bioconductor version: Release (3.17). https://doi.org/10.18129/B9.bioc.arrayQualityMetrics.

Klaus, Bernd, and Stefanie Reisenauer. 2018. “An End to End Workflow for Differential Gene Expression Using Affymetrix Microarrays.” F1000Research. https://doi.org/10.12688/f1000research.8967.2.

Kruskal, William H, and W Allen Wallis. 1952. “Use of Ranks in One-Criterion Variance Analysis.” *Journal of the American Statistical Association* 47 (260): 583–621.

Lex, Alexander, Nils Gehlenborg, Hendrik Strobelt, Romain Vuillemot, and Hanspeter Pfister. 2014. “UpSet: Visualization of Intersecting Sets.” *IEEE Transactions on Visualization and Computer Graphics* 20 (12): 1983–92. https://doi.org/10.1109/TVCG.2014.2346248.

Mahmoud, Ranim, Virginia Kimonis, and Merlin G. Butler. 2022. “Genetics of Obesity in Humans: A Clinical Review.” *International Journal of Molecular Sciences* 23 (19): 11005. https://doi.org/10.3390/ijms231911005.

Patil, Indrajeet. 2021. “Visualizations with Statistical Details: The ‘ggstatsplot’ Approach.” *Journal of Open Source Software* 6 (61): 3167. https://doi.org/10.21105/joss.03167.

Rebhan, M., V. Chalifa-Caspi, J. Prilusky, and D. Lancet. 1997. “GeneCards: Integrating Information about Genes, Proteins and Diseases.” *Trends in Genetics: TIG* 13 (4): 163. https://doi.org/10.1016/s0168-9525(97)01103-7.

Ritchie, Matthew E., Belinda Phipson, Di Wu, Yifang Hu, Charity W. Law, Wei Shi, and Gordon K. Smyth. 2015. “Limma Powers Differential Expression Analyses for RNA-Sequencing and Microarray Studies.” *Nucleic Acids Research* 43 (7): e47. https://doi.org/10.1093/nar/gkv007.

Schwarz, Gideon. 1978. “Estimating the Dimension of a Model.” *The Annals of Statistics* 6 (2): 461–64. https://doi.org/10.1214/aos/1176344136.

SHAPIRO, S. S., and M. B. WILK. 1965. “An Analysis of Variance Test for Normality (Complete Samples)†.” *Biometrika* 52 (3–4): 591–611. https://doi.org/10.1093/biomet/52.3-4.591.

Smyth, Gordon K. 2004. “Linear Models and Empirical Bayes Methods for Assessing Differential Expression in Microarray Experiments.” *Statistical Applications in Genetics and Molecular Biology* 3:Article3. https://doi.org/10.2202/1544-6115.1027.

Tjørve, Kathleen M. C., and Even Tjørve. 2017. “The Use of Gompertz Models in Growth Analyses, and New Gompertz-Model Approach: An Addition to the Unified-Richards Family.” *PLOS ONE* 12 (6): e0178691. https://doi.org/10.1371/journal.pone.0178691.
